# Kinetic oxygen measurements by CVC96 in L-929 cell cultures

**DOI:** 10.1186/1746-160X-2-6

**Published:** 2006-03-01

**Authors:** Ulrich Plate, Tobias Polifke, Dieter Sommer, Jörg Wünnenberg, Hans-Peter Wiesmann

**Affiliations:** 1 Department of Cranio-Maxillofacial Surgery, University of Münster, Waldeyerstraße 30, D-48129 Münster, Germany; 2 O2-SCAN GmbH, Mendelstr. 7, D-48149 Münster, Germany

## Abstract

Generally animal and human cells use oxygen during their whole life. Consequently the oxygen use is a simple indicator to test the vitality of cells. When the vitality decreases by the delivery of toxic substances the decrease can be observed directly by the oxygen-use of the cells. To get fast information of the vitality of cells we have measured the O_2_-tension by testing a new model of a bioreactor, the Cell Vitality Checker 96 (CVC96), in practical application. With this CVC96, soon a simple test will exist for the measurement of the oxygen use. In this respect the question had to be answered whether the use in the laboratory is easy and whether oxygen as a parameter in the vitality test can also be applied in future for problems in the field of material testing.

## Background

A recent challenge to bone and cartilage tissue engineering is to lift up research-scale products to a level of reproducible tissue substitute fabrication that is clinically effective, e.g. by the use of bioreactors [1,2]. Bioreactors can be considered as devices in which biological and/or biochemical processes are performed under controlled conditions (e.g. pH, temperature, pressure, oxygen supply, nutrient supply and waste removal). Most of the bioreactors were initially developed to test biomaterials [3]. The survival of cells in vivo as well as in bioreactors depends on the response of the distinct cells to the environment. A higher oxygen tension for example is needed for osteoblastic differentiation, whereas prolonged hypoxia favours formation of cartilage or fibrous tissue [4]. The adjustment of oxygen tension in bioreactors is therefore a critical aspect in bioreactor design. The simplest and most widely used bioreactor for bone and cartilage tissue engineering today is indeed the culture dish [5]. Animal and human cells use oxygen during their whole life. Consequently the oxygen use is a simple indicator to test the vitality of cells.

Vitality and cytotoxicity tests are established techniques in different fields such as cell culture control, search of active agents and also in other medical techniques. To the broad field of medical technology belongs also the testing of biocompatibility in the field of development and examination of tooth- and bone replacement tissues in cranio-maxilliofacial surgery. The research group for biomineralization and tissue engineering of this department carries out the testing of biocompatibility.

## Methods

**Figure 1:**
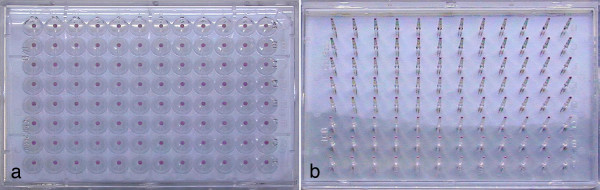
Front (a) and back (b) side of the CVC96-lid.

Up to now the temporal course of cell reactions on toxic influences could be observed only with a high input of labor time (and costs). Because of this high input we have tested the new measuring technology CVC96 with a short test in practical applications. CVC96 allows the possibility to observe and evaluate cell tests also kinetically. This new field of vitality tests was the reason for a fundamental application test in our research unit. CVC96 is a lid for 96-well cell culture plates, which is used instead of the standard lid. CVC96 has a pin for each well, which is placed in the middle of a well (Fig. 1a,b).  This means, the pins are dipped in the cell culture medium. The pin is in the medium without any contact to the cells at the bottom of the well. The top of each pin is covered by a gas permeable membrane. Within this membrane a fluorescent dye is embedded, which shows its fluorescence in dependence of the O_2_-tension. Thus CVC96 shows O_2_-dependent signals without contact to cells or irritation of cells. In this case a simple conventional fluorescence-reader for micro titer plates is sufficient for read out after incubation. During complete measurements there is no contact of pin or fluorescent dye to the cells and no chemicals have to be added, thus saving the procedure of pipetting. Since the cells are not influenced by the measurement, the measurements can be repeated at anytime and thus a kinetic profile can be registered. This is not possible with standard vitality tests, which are end-point assays. Thus it is possible to get more relevant data than up to now, since also the speed of cellular responses can be determined directly. Further, a definite evaluation of a test experiment is often possible many hours earlier, because the "end point" of an assay doesn't need to be awaited. The new test can be controlled during its course and stopped as soon as a clear result is received. Since the cells are not consumed or affected during the test, further analyses with the same cells are possible if needed. For instance after a test experiment also used cell media can be taken in order to determine, after corresponding analysis, rates of uptake of sublethal toxic material, because the cells remain intact and contain absorbed substances. A microscopical control of the cells after the assay is also possible. The effect on the vitality of the cells inclusively can be received visually and correlated in a single assay. The practical test was carried out with CVC96, kindly provided by the producer Sanochemia Diagnostics from an early product series. The analysis of a general usefulness in a laboratory was a fundamental point of interest. It was the goal to find out whether usage and measurement would be easy. A further question was whether the quality and plausibility of the received data would be convincing. Finally the question in our research unit should be answered, whether our research project could use this assay in future. The experiments were carried out in L-929 cell cultures (Tab.1) [6-9].

**Table 1:** DSMZ Cell Culture Data for L-929 cells.

Cell line:	L-929
Cell type:	mouse connective tissue fibroblast
Origin:	established from the normal subcutaneous areola and adipose tissue of a male C3H/An mouse; used as target in TNF detection assays
Morphology:	fibroblasts growing as monolayer
Medium:	90% RPMI 1640 + 10% FBS
Subculture:	split confluent cultures 1:5 to 1:10 using trypsin (do not use trypsin/EDTA); after 2–3 days monolayer will be confluent; split 2–3 times a week; seed out initially at about 1.0–2.0 × 10^6 ^cells/25 cm^2^
Incubation:	at 37°C with 5% CO_2_
Doubling time:	ca. 21–24 hours
Harvest:	ca. 4–8 × 10^6 ^cells/25 cm^2^
Storage:	frozen with 70% medium, 20% FBS, 10% DMSO at about 1–2 × 10^6 ^cells/ampoule
Mycoplasma:	negative in DAPI, microbiological culture, PCR assays
Species:	confirmed as mouse with IEF of AST, MDH, PEP B
Cytogenetics:	murine hypertriploid karyotype – 61–67, 12–16 centric fusion markers present
Viruses:	ELISA: reverse transcriptase negative

The L-929 cell cultures were seeded in 96-wells. After becoming confluent, the cells were treated with three different types of media: a) media that had been incubated for 10 days with PMMA (poly-methyl-methacrylate), a preferable inert material for applications in medical technology, b) media that had been incubated for ten days with a collagen matrix, a material known to support the growth of bone cells. Besides the differently treated cells also untreated cells were measured as a reference. Further glutaraldehyde, a toxic chemical substance, was added to the L-929 cell cultures in different concentrations (0.1%, 1.0%, 10%). The cells were kept at 37°C in CO_2_-incubators during the whole experimental time and were removed from the incubator only for measurements. The measurements were carried out in a fluorescence-reader, FLX800T instrument from Bio-Tek Instruments GmbH, with which the data could be read in discontinuous kinetic files. The same modified media with PMMA and collagen were also tested for cell vitality in an identical attempt with following evaluation by a Resazurin-assay (CCS-PRINCESS^® ^CELIA Instant Cytotoxicity-Assay 2004).

## Results and discussion

**Figure 2:**
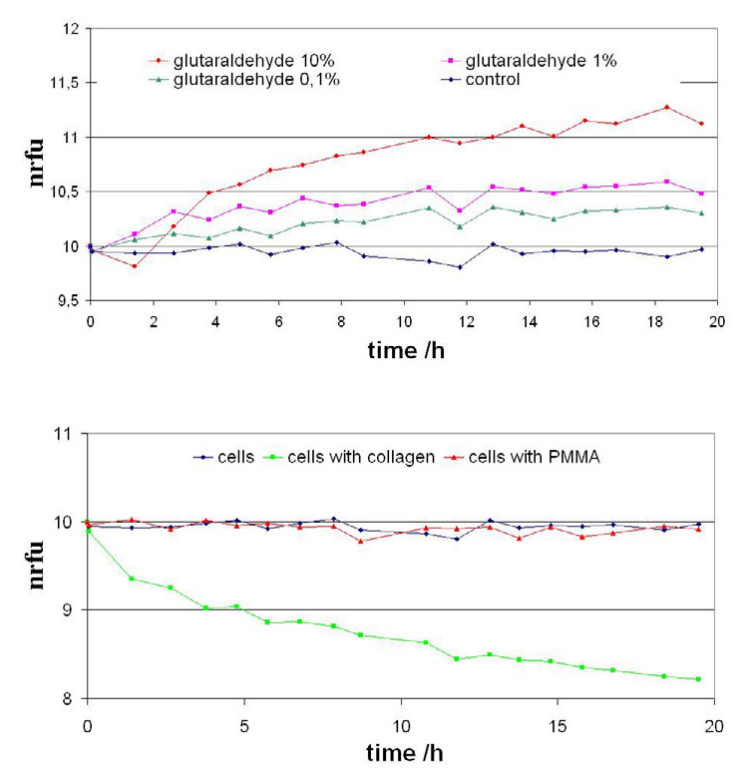
Measuring curves of the kinetic course of the oxygen-signals [measured Δ fluorescence in normalized relative fluorescence units (nrfu)] from L-929-cells incubated with media, (top) which had been treated with different cytotoxic glutaraldehyde concentrations (0.1%,1%,10%) and (bottom) modified media by PMMA and by collagen matrix for 20 h, respectively.

Living human and animal cells need and use oxygen, so that the culture medium with living cells contains relatively less oxygen than the surrounding atmosphere. Continuously oxygen diffuses into the cell culture medium. Glutaraldehyde induces the dying of cells and therefore the consumption of oxygen by living cells decreases and the share of oxygen in the cell medium increases (Fig. 2, top). Not only clear results evolve, which reflect the row of different concentrations of the toxic substance glutaraldehyde, but furthermore this kinetic registration is the basis for further future information. A clear correct result of those assays would result already in 5–10 hours. So working time to get first useable results can be saved in this kinetic experiment. Besides the principle suitability of the tests for the clarification of cytotoxic effects it is also very important for clinical applications to know the applicability of this covers to test clinical materials, here PMMA and collagen matrix (Fig. 2, bottom). These two substances were incubated with cell medium whereby the influence of the two substances on cells is sufficiently known, and both are used in dental medical research and application. The manipulated media were sterilized again, respectively and used as the cell media in the following CVC96 experiments. The measurement of cell oxygen consumption is a sufficient tool for characterization of cell promotion or an impaired cell activity by biomaterials *in vitro*.

The differences between the influence of collagen matrix, which showed as to expected a clear stimulating effect on the cells and that of PMMA, which showed no differences to the untreated medium, is clearly visible (Fig. 2, bottom). Also for this question clear results can be observed after 5 to 10 hours.

**Figure 3:**
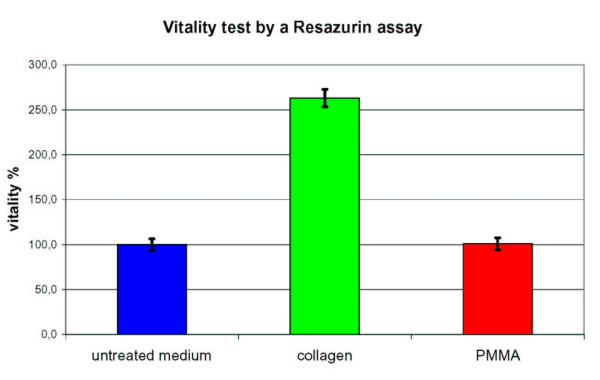
Resazurin-Assay for a comparison of vitality of L-929-cells incubated with medium, modifed by PMMA and by collagen matrix for 20 h (n = 8) (PRINCESS^® ^CELIA Assay of CCS, Hamburg).

Comparing measurements by a Resazurin assay show identical tendencies (Fig. 3). For this first practical test of CVC96, the Resazurin assay has been done only with n = 8, because effects of PMMA and collagen are well known [10-13]. While the demoted PMMA-medium shows identical vitality to the untreated medium a clear stimulation can be observed with collagen matrix. Since the Resazurin-assay shows a consuming "end-point"-determination only one measuring point after 20 h for incubation is registered.

The usage of the new CVC96-assays is easier in relation to the standard assays, because the pipetting work steps are not necessary. The quality of the data and their reliability increase by kinetic evaluation. The experiments can be carried out in shorter time, because in many cases no standardized temporal end-point of the measurement has to be awaited.

## Conclusion

Up to now only the results of first praxis tests could be shown. Surpassing this first practical test in future, the validity of the data in relation to standard tests must be intensively investigated so that afterwards fundamental questions of biocompatibility can be investigated for pure and mixed materials. We regard CVC96 as an interesting assay which allows new possibilities of investigations on the basis of kinetic observations and gives new insights and faster results. Thus it should be extensively validated after being brought onto the market by the producer. This will include statistical relevant testing with different kind of cells and a greater number n of tested material.

## Competing interests

The authors declare that they have no competing interests. O2-SCAN GmbH has no financial interest in this study.

## Authors' contributions

HPW and UP have designed and managed the study. TP has analyzed the data. DS has prepared the cell cultures and the probes. JW has measured the fluorescent units. All authors have contributed to the manuscript preparation.

## References

[B1] Salgado AJ, Coutinho OP, Reis RL (2004). Bone tissue engineering: state of the art and future trends. Macromol Biosci.

[B2] Martin I, Wendt D, Heberer M (2004). The role of bioreactors in tissue engineering. Trends Biotechnol.

[B3] Meyer U, Szulczewski HD, Möller K, Heide H, Jones DB (1993). Attachment kinetics and differentiation of osteoblasts on different biomaterials. Cells Mater.

[B4] Malda J, Martens DE, Tramper J, Van Blitterswijk CA, Riesle J (2003). Cartilage tissue engineering: controversy in the effect of oxygen. Crit Rev Biotechnol.

[B5] Langer R, Vacanti JP (1993). Tissue engineering. Science.

[B6] Suzuki T, Ohashi R, Yokogawa Y, Nishizawa K, Nagata F, Kawamoto Y, Kameyama T, Toriyama M (1999). Initial anchoring and proliferation of fibroblast L-929 cells on unstable surface of calcium phosphate ceramics. J Biosci Bioeng.

[B7] Suzuki T, Hukkanen M, Ohashi R, Yokogawa Y, Nishizawa K, Nagata F, Buttery L, Polak J (2000). Growth and adhesion of osteoblast-like cells derived from neonatal rat calvaria on calcium phosphate ceramics. J Biosci Bioeng.

[B8] Siggelkow W, Gescher DM, Siggelkow A, Klee D, Malik E, Rath W, Faridi A (2004). In vitro analysis of modified surfaces of silicone breast implants. Int J Artif Organs.

[B9] Korematsu A, Furuzono T, Yasuda S, Tanaka J, Kishida A (2005). Nano-scaled hydroxyapatite/polymer composite III. Coating of sintered hydroxyapatite particles on poly(4-methacryloyloxyethyl trimellitate anhydride)-grafted silk fibroin fibers. J Mater Sci Mater Med.

[B10] Knapp HF, Reilly GC, Stemmer A, Niederer P, Knothe Tate ML (2002). Development of preparation methods for and insights obtained from atomic force microscopy of fluid spaces in cortical bone. Scanning.

[B11] Ohsawa K, Neo M, Matsuoka H, Akiyama H, Ito H, Nakamura T (2001). Tissue responses around polymethylmethacrylate particles implanted into bone: analysis of expression of bone matrix protein mRNAs by in situ hybridization. J Biomed Mater Res.

[B12] Dard M, Sewing A, Meyer J, Verrier S, Roessler S, Scharnweber D (2000). Tools for tissue engineering of mineralized oral structures. Clin Oral Investig.

[B13] Zambonin G, Colucci S, Cantatore F, Grano M (1998). Response of human osteoblasts to polymethylmetacrylate In vitro. Calcif Tissue Int.

